# Stigmatization and Interpersonal Deviance Behaviors of Tour Guides: The Influence of Self-Identity Threat and Moral Disengagement

**DOI:** 10.3389/fpsyg.2022.765098

**Published:** 2022-02-28

**Authors:** Aimin Deng, Wenxing Liu, Anna Long, Yanghao Zhu, Kai Gao

**Affiliations:** ^1^Tourism Management, School of Business Administration, Zhongnan University of Economics and Law, Wuhan, China; ^2^Human Resource Management, School of Business Administration, Zhongnan University of Economics and Law, Wuhan, China

**Keywords:** tour guide stigmatization, interpersonal deviance behavior, self-identity threat, moral disengagement, tourism

## Abstract

Severe tour guide stigma is a significant problem hindering tourism development. Based on self-identity threat and moral disengagement theory, this study analyzed the relationship between tour guide stigmatization and tour guides’ interpersonal deviance behavior. Survey data collected from 241 tour guides at three different points in time showed that tour guide stigmatization was positively related to tour guides’ interpersonal deviance behavior and that self-identity threat mediated this effect. The results also show that moral disengagement moderated the effect of tour guides’ self-identity threat on interpersonal deviance behavior, as well as the indirect effect of tour guide stigmatization on tour guides’ interpersonal deviance behavior *via* self-identity threat. This study enriches theoretical research on tour guide stigmatization and offers practical suggestions for solving stigmatization problems for tour guides and organizations.

## Introduction

As an essential part of the tourism industry, tour guides are the vital link between destinations and tourists (Wong and Wang, 2009). The quality of service of tour guides is a key factor affecting tourists’ satisfaction, loyalty, and word-of-mouth communication (Mossberg, 1995). Recently, negative service behaviors, such as fraud, intimidation, and threats to tourists, on the part of tour guides, have become common (Wong and Lee, 2012). This situation reduces tourists’ satisfaction and deepens the public’s negative impression of tour guides. According to a report by the Travel Quality Assurance Association in Taiwan (2020), tour guides ranked second from last in the image of each profession. “Unscrupulous” and “blinded by greed” have become stereotypes of tour guides as perceived by tourists. More and more tourists said that they no longer trusted tour guides and would not choose them to provide services in future trips (Galí and Aulet, 2019). Tour guides have been belittled and ostracized by the public and have even become objects of ridicule and contempt (Larsen and Meged, 2013). Thus, the profession of tour guide has become stigmatized (Stafford and Scott, 1986). In this context, it is unclear to what extent and how tour guide stigma influences tour guide behaviors.

Stigma is a negative evaluation caused by the negative attribution of stereotypes, and negative stereotypes about employees cause occupational stigma (Pilgrim, 2011). Ashforth and Kreiner (1999) identified three different sources of occupational stigma: physical, social, and moral. Research in the field of occupational stigma usually focuses on physical or social stigma. Therefore, the definition of occupational stigma is also related to physical or social stigma. Vlijmen (2019) defined the physical occupational stigma of cleaners as the stigma caused by physical contact with dirt during the working period (i.e., dealing with garbage). The definition of social occupational stigma is related to social contact that is considered dirty, such as the work of prison guards that requires contact with prisoners (Eriksson, 2021). Compared with physically or socially stigmatized occupations, morally stigmatized occupations result not from contact with dirt but from behavior that is considered dirty and employees in these jobs usually have more additional social resources to confront the stigma (Ashforth and Kreiner, 2014). The stigma effect on morally stigmatized occupations may differ among stigmatized occupations. According to Li et al. (2021), tour guides’ stigma is due to their unethical behaviors (e.g., deceiving tourists or forcing them to shop), implying that tour guide stigma is primarily a moral occupational stigma. Therefore, drawing from occupational stigma studies that consider the nature of the behavior giving rise to the stigma, tour guide stigma is defined in this study as the negative evaluation of this occupation formed by tourists due to the moral corruption of individual tour guides.

Some studies on stigma show that practitioners who perceive stigma may resist it (Link and Phelan, 2001) through a series of interpersonal deviance behaviors (Bennett and Robinson, 2000), such as violating and harming their clients (Shantz and Booth, 2014). Therefore, resistance is incapable of eliminating the stigma; it may aggravate it further. Significantly, in the information age, the wide use of social media has expanded the scope and speed of behavior transmission (Schweitzer, 2014), thereby forming a vicious circle of “interpersonal deviance behavior–stigmatization–interpersonal deviance behavior.” However, in previous studies, the focus of tour guide interpersonal deviance behavior was mostly on salary (Hsieh and Wu, 2007) and welfare (Hu and Wall, 2013). There is a lack of research on the influence mechanism of tour guide stigmatization from the perspective of interpersonal interaction, thereby limiting our understanding of the implications of tour guide stigmatization for interpersonal deviance behavior. Therefore, to contribute to the tour guide stigmatization literature and extend theoretical knowledge regarding the effect of tour guide stigmatization on interpersonal deviance behavior and its underlying mechanisms, it is important to investigate this neglected research question.

According to identity threat theory (Petriglieri, 2011), although individuals have multiple identities (Markus and Wurf, 1987), individuals will experience self-identity threat when one of their identities becomes devalued (Dutton et al., 2010), it loses its meaning (Hall, 2002), is no longer presentable (Rothbard, 2001), or is potentially harmful (Anteby, 2008). The degree of self-identity threat perception causes different interpersonal deviance behaviors (Murtagh et al., 2012). Therefore, drawing on identity threat theory, we selected self-identity threat as the mediator and posited that tour guide stigmatization would cause interpersonal deviance behavior by producing self-identity threat. Furthermore, moral disengagement theory (Bandura, 1985) points out that, as a cognitive tendency, moral disengagement affects the identification of the resulting behavior by transferring harmful behaviors to behaviors that are acceptable for individuals and the public, thereby generating interpersonal deviance behavior. Moral disengagement theory suggests that moral disengagement may serve as a moderator that influences whether a self-identity threat enhances interpersonal deviance behavior. In short, the current study draws on self-identity threat and moral disengagement theory, proposing a moderated mediation model in which tour guide stigmatization promotes interpersonal deviance behavior *via* self-identity threat. It is also proposed that such an indirect effect is contingent on moral disengagement.

This manuscript contributes to the literature on tour guide stigmatization in several ways. First, we expanded the research on the consequences of tour guide stigmatization. When tour guides perceive stigmatization, they may engage in deviance behavior toward tourists. Second, self-identity threat has been shown to mediate tour guide stigmatization and tour guide interpersonal deviance behavior. By revealing the mediating role of self-identity threat, we can provide theoretical and practical implications for interventions that curb the interpersonal deviance behavior of tour guides. Third, we explore the boundary conditions of moral disengagement in the effect of self-identity threat on the interpersonal deviance behavior of tour guides. Our findings require tourism organizations to focus on the moral level of tour guides to reduce their interpersonal deviance behavior.

## Literature Review and Hypotheses

### Tour Guide Stigmatization

Stigmatization is the act of giving something a negative label, vilifying a person or a group because of stigmatized characteristics, and causing individuals not to be accepted and recognized by others (Goffman, 1963). Based on the background causes of their formation, there are three types of stigma: physical, social, and moral. Ashforth and Kreiner (1999) provided criteria for each of the three forms of stigma. The authors believe that physical stigma occurs when an occupation is either directly associated with garbage, with death, or is performed under noxious or dangerous conditions. Social stigma is applied to occupations that bring individuals into contact with people or groups who are considered stigmatized. Moral stigma occurs when employees are deemed to use methods that are deceptive, intrusive, confrontational, or violate civilized norms. A tour guide could be described as a leader (Tsaur and Teng, 2017) who provides tourists with information about the local landscape, acts as an interpreter, and offers other related services during the tour journey. During their service, tour guides spend the longest contact time with tourists, and their service performance can directly affect tourists’ evaluations of a destination (Huang et al., 2010). In the past, tour guides had a high status and a positive occupational image because they were a necessary link between destinations and tourists (Wong and Wang, 2009). They were also well-paid (Mak et al., 2011). However, in recent years, the working environment of tour guides has involved income instability (Min, 2014), unlimited working hours (Hsieh and Wu, 2007), and insufficient welfare provision (Ap and Wong, 2001). In order to survive and have some free time (Min, 2014; Lu et al., 2016), more and more tour guides began to engage in improper behavior, such as earning commissions from tourists’ shopping and from their entertainment consumption. Tourists stated that they had bought products under pressure from tour guides (Wong and Lee, 2012), and they accused by tour guides of spending too little money themselves. As the role of tour guide transformed from that of an interpreter to that of a sales adviser, when tourists did not buy or consume anything, the tour guide insulted and ridiculed them, resulting in increasing conflicts. According to the Chinese Tourism 3.15 Complaint Platform (2019), the number of complaints against tour guides was in the top five list of consumer complaints from 2015 to 2020. In the eyes of tourists, tour guides have become greedy and dishonest (Dahles and Bras, 1999). In short, we can further define tour guide stigmatization as a behavior arising from the moral failure of tour guides, causing tourists to consider their profession in a derogatory and insulting manner.

### Tour Guide Stigmatization and Interpersonal Deviance Behavior

The negative impact of stigmatization on tour guides and the development of the tourism industry are far-reaching. From the perspective of tour guides, stigmatization causes them to have a high negative perception of their occupation (Ashforth and Kreiner, 2014). They also experience the pressure of stigma management (Baran et al., 2012). Bove and Pervan (2013) found that coping with stigma usually consumes the practitioner’s resources. If the resources consumed by stigma management are greater than the potential benefits gained from the job, practitioners face increased pressure. The declining professional identity of tour guides and the tremendous psychological pressure experienced by tour guides cause a high turnover rate in the tour guide profession. This situation is not conducive to the sustainable and healthy development of tour guide teams. From the perspective of the tourism industry, tour guides are front-line service providers who face the praise or criticism of tourists. The image of tour guides affects tourists’ perceptions of other tourism practitioners (Huang et al., 2010). In interactions with tourists, when a tour guide perceives and resists stigma, conflict with tourists can occur, thereby aggravating the tour guide stigma. Therefore, the consequences of a tour guide’s behavior in dealing with stigma not only affect the image of the tour guide but also affect the image of the tourist destination, the tourism enterprises, and the other practitioners in the tourism industry.

Interpersonal deviance behavior refers to behavior that violates the norms and expectations of social groups (Kaplan and Lin, 2000). In interpersonal communication, interpersonal deviance behavior usually takes the form of lying, cheating, insulting, speaking badly, mocking, slandering, threatening, and other behaviors that are offensive to others (Bennett and Robinson, 2000). In serious cases, it might even intentionally hurt others. Relevant research shows that interpersonal deviance behavior is the result of the combined effects of society and individual psychology. When the social environment is hostile to personal development, individuals might have a negative prediction of the social environment and adopt a negative coping style in interpersonal communication. This might be accompanied by aggressive behavior. Occupational stigmatization is a form of social hostility toward a particular occupation. Levin and Van Laar (2006) found that when individuals perceive that their occupation is stigmatized, they tend to behave aggressively with clients who stigmatize them. It could be inferred that when tour guides perceive that society is hostile to their profession (professional stigma), they feel the need to rebalance the situation by acting in impolite and unfriendly ways. For the above reasons, we hypothesize the following:

Hypothesis 1. Tour guide stigmatization is positively related to tour guides’ interpersonal deviance behavior.

### The Mediating Role of Self-Identity Threat

Identity threat theory typically includes two stages of self-identity threat generation and response (Petriglieri, 2011). This theory proposes that individuals have multiple identities in society and maintain their sense of self-worth through these identities, aiming to seek meaning for their existence in society (Reed, 2004). This is the basic premise of self-identity threat. In general, self-identity is a stable state in a particular environment, and individuals have a sense of adaptation, continuity, and meaning (Erikson, 1968). However, when negative social events or evaluations occur, the individual perceives that certain factors prevent affirmation or a display of self-identity (Elsbach, 2003). The result can be a tendency to fall into self-doubt and to believe that the individual is threatened to some extent. Generally speaking, these factors include the classification of individuals against their will, the hindrance and destruction of group uniqueness, the destruction of group values, and the destruction of the individual’s position in the group. In social interactions, stigmatized individuals feel shame because their stigmatized characteristics damage their original self-perception. When self-identity is damaged, a self-identity threat arises (Breakwell, 1986). For example, in a study of weight stigma, people who defined themselves as obese tended to be concerned about their partner’s evaluation and had low self-perception. They were constantly afraid that their excess weight would cause their partners to abandon them (Boyes and Latner, 2009). Occupational stigmatization is one of the concrete manifestations of stigma in society. It is a phenomenon in which a specific profession is devalued by society or, under certain circumstances, is labeled as having unwelcome characteristics (Goffman, 1969). Whether willing or not, individuals who engage in stigmatized occupations are likely to self-define based on occupational stigma characteristics and perceive the potential harm that such stigma could cause to their social identity, resulting in self-identity threat (Major and O’Brien, 2004). Therefore, we propose the following:

Hypothesis 2. Tour guide stigmatization is positively related to tour guides’ self-identity threat.

Identity threat theory points out that when a self-identity threat is generated, individuals engage in coping methods in an attempt to alleviate anxiety. In other words, in the face of a self-identity threat, to rebuild their integrity, individuals usually seek out strategies to restore their social identity value and their sense of belonging. There are three common strategies. One is the avoidance and withdrawal strategy. This behavior is seen as the primary strategy used to remove the self-identity threat. Individuals maintain their self-worth and meaning by moving away from a threatening environment. In the field of consumption, Branscombe and Wann (1994) found that consumers faced with self-identity threat might aim to maintain their self-identity by avoiding the connection between themselves and certain disadvantaged groups by deliberately avoiding certain consumer goods. In workplace studies, Trevor and Nyberg (2008) found that layoffs increased employees’ self-identity threat and led to an increase in the voluntary resignation rate. The second strategy involves repositioning, the process whereby the individual reshapes positive self-concept to the greatest possible extent. It is possible to maintain cumulative self-awareness by reducing the aspect of comparison to selected groups that are in a worse position than oneself in a certain respect (Ashforth and Kreiner, 1999) or to introduce some new dimension of comparison to bring about positive differentiation (Wang and Liu, 2007). The last strategy is counterattack, the process of directly confronting the dominant group to eliminate the self-threat. Fischer et al. (2010), in a study of individual aggressive behavior and identity threat, found that self-identity threat was an important reason for individuals to engage in aggressive and retaliatory behavior. This means that individuals confronted with self-identity threat are likely to exhibit aggressive, retaliatory, and harmful deviance behaviors. Based on the counterattack strategy in self-identity threat theory, we propose the following:

Hypothesis 3. Tour guide self-identity threat is positively related to tour guides’ interpersonal deviance behavior.

In sum, stigmatization will not only lead directly to interpersonal deviance behavior but it will also produce self-identity threat. Considering that self-identity threat is also an important cause of interpersonal deviance behavior, we further propose:

Hypothesis 4. The positive impact of tour guide stigmatization on tour guides’ interpersonal deviance behavior is partly mediated by self-identity threat.

### The Moderating Role of Moral Disengagement

Bandura (1999) defined moral disengagement as the process by which individuals excused their immoral deviance behavior cognitively, aiming to reconstruct non-moral deviance behavior into moral behavior and ignored or distorted the consequences of the behavior, thereby minimizing their responsibility for the consequences. Specifically, moral disengagement can be divided into eight mechanisms: moral justification, euphemistic labeling, advantageous comparison, displacement of responsibility, diffusion of responsibility, distortion of consequences, dehumanization, and attribution of blame (Bandura, 1985). Among the mechanisms, moral justification, euphemistic labeling, and advantageous comparison could allow individuals to compare their deviance behavior with more serious behaviors and to use language modification to reinterpret and rationalize their deviance behavior. Displacement of responsibility, diffusion of responsibility, and distortion of consequences aim to minimize the consequences of interpersonal deviance behavior by individuals’ shirking responsibility. When harmful results are ignored, minimized, or distorted, individuals have little reason to condemn themselves and not to engage in deviance behavior (Bandura et al., 1996). Dehumanization is the process of removing or weakening self-condemnation by depriving individuals of their humanity or by endowing them with dehumanizing characteristics. Attribution of blame is a process that ignores moral issues and considers deviance behaviors as being forced by others or by the environment.

In general, when moral self-regulation functions normally, individuals can recognize that their behavior conflicts with moral standards, and they can prevent deviance behavior by self-condemnation. When moral disengagement is introduced, individuals’ ability for moral self-regulation is affected. Therefore, the cognitive connection between deviance behavior and self-punishment fails, resulting in more deviance behaviors (Moore et al., 2012). For example, studies on prison bullying have shown that prisoners often participate in prison gangs and bully other vulnerable prisoners based on advantageous comparison mechanisms. The higher the level of moral disengagement, the more obvious this phenomenon becomes (Wood et al., 2009). In tobacco enterprises, to reduce responsibilities and maintain a good image, the enterprise usually adopts responsibility displacement and diffusion to achieve moral disengagement. Strategies include decentralizing responsibility for product production and assigning responsibility to technicians, operators, sale representatives, and exporters. Therefore, social cognitive theory research generally shows that an individual’s level of moral disengagement is an important factor driving deviance behaviors (Douglas, 1995). The higher the level of moral disengagement, the more severely the self-condemnation is weakened, and the greater the possibility of deviance behavior. Conversely, when individuals have a low level of moral disengagement, their moral self-regulation ability is stronger, and they can clearly recognize the harmfulness of deviance behavior. Strong self-condemnation restrains the impulse to engage in deviance behavior. In short, as a cognitive tendency, moral disengagement has the effect of moderating the relationship between identity and behavior. Hence, we propose the following:

Hypothesis 5. The positive relationship between tour guide self-identity threat and tour guides’ interpersonal deviance behavior is moderated by moral disengagement such that the relationship is stronger (vs. weaker) for higher (vs. lower) moral disengagement.

Hypothesis 4 points out the mediating effect of self-identity threat on tour guide stigmatization and tour guides’ interpersonal deviance behavior. Hypothesis 5 proposes that the influence of self-identity threat on tour guides’ interpersonal deviance behavior is different for different degrees of moral disengagement. Therefore, we propose the following moderated mediation hypothesis:

Hypothesis 6. The positive indirect effect tour guide stigmatization has on tour guides’ interpersonal deviance behavior *via* self-identity threat is moderated by moral disengagement such that the indirect relationship is stronger (vs. weaker) for higher (vs. lower) moral disengagement.

**FIGURE 1:**
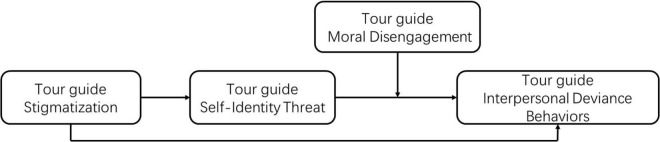
Research model.

To sum up, we propose the research model (see Figure 1).

## Sample and Procedures

### Resource Identification Initiative

Because this study concerns tour guide stigmatization, it was difficult to contact the target participants (tour guides with qualification certificates) through random sampling. Therefore, we relied on tour guide associations representing 1,439 tour guides from 18 travel agencies to distribute our questionnaires. The data collection took place during the COVID-19 pandemic. Therefore, to minimize unnecessary interpersonal contact, we used electronic surveys. Previous studies have indicated that data collected through electronic surveys are reliable and have the advantage of recruiting more participants (Chen and Eyoun, 2021). To maximize response rates, the respondents were paid $3.00 for their anonymous and voluntary participation. All the participants in this study had to complete the tour guide stigmatization, self-identity threat, interpersonal deviance behavior, and moral disengagement scales. They also had to supply their demographic information.

Data were collected in three data phases, with a 1-month interval between each phase. In the first phase, tour guides completed a questionnaire that included the tour guide stigmatization scale and control variables. In the second phase, the questionnaire included the self-identity threat scale and the moral disengagement scale. In the last phase, only the interpersonal deviance behavior scale was included. A total of 241 valid questionnaires were collected from the same group of personnel for the three measurements. The sample size was five times greater than the number of measurement items, thereby meeting the requirements of the relevant studies (Bentler, 1989).

Among the 241 tour guides, 48.5% were men, aged 18–56 (*M* = 33.87), 5.4% had junior high school level or below, 80.9% had senior high school level, and 13.7% had bachelor’s degrees or higher. From a business scope point of view, 8.7% of the tour guides were employed overseas. Of the remaining 91.3%, 20.9% stay with groups for their entire trip, while 79.1% only provide services at tourist destinations (12% are only responsible for particular scenic tourist attractions). Of the tour guides, 74.3% are full-time, 12.4% have been working as tour guides for less than 1 year, 31.5% have been working as tour guides for between 1 and 5 years, 49% for between 5 and 10 years, and 7.1% for more than 10 years. In terms of professional status, 69.3% were junior, 17.4% were intermediate, 10% were senior, and 3.3% were special. The monthly income of 38.2% of the tour guides is less than 5,000¥. The monthly income of 40.6% of them is between 5,000¥ and 10,000¥, and 21.2% of them earn more than 10,000¥ a month. Regarding the characteristics of the respondents, the sample distribution of this study is similar to that Tuniu (2020).

### Measures

In this study, we measured tour guide stigmatization, self-identity threat, interpersonal deviance behavior, and moral disengagement using a 5-point Likert scale (1 = “strongly disagree,” 5 = “strongly agree”).

#### Tour Guide Stigmatization

The tour guide stigmatization scale in this study is based on the Shantz and Booth (2014) call center staff stigmatization scale. This scale has six items, such as “Most people who are not tour guides have a lot more negative thoughts about tour guides than they actually express.” Cronbach’s alpha was 0.811.

#### Self-Identity Threat

The four-item scale developed by Breakwell (1988) was used to assess self-identity threat. In line with the research object, this manuscript adjusted the four items. For example, “Negative impressions of tour guides undermine my sense of self-worth.” Cronbach’s alpha was 0.778.

#### Interpersonal Deviance Behavior

To measure tour guides’ interpersonal deviance behavior, we selected the deviance behavior scale compiled by Bennett and Robinson (2000). The scale mainly includes two dimensions: interpersonal deviance and organizational deviance. The interpersonal deviance behaviors scale has seven items. Sample items included “Made fun of tourists at work.” Cronbach’s alpha was 0.844.

#### Moral Disengagement

Moral disengagement was measured using Moore et al. (2012) eight-item scale. Sample items included: “It is okay to spread rumors to defend those you care about.” Cronbach’s alpha was 0.806.

#### Control Variables

In line with previous research, in addition to selecting the guide’s gender, age, education, and tenure as control variables (Chang, 2014), we selected business scope, occupational attributes, occupational level, and income as other control variables.

### Common Method Variance Analysis

Before data analysis, to ensure the accuracy of the evaluation results, it was necessary to check for common method deviations in the questionnaire. The commonly used method is Harman’s single-factor test (Karatepe, 2010). We used SPSS 23.0 to conduct a non-rotating exploratory factor analysis on all the items of tour guide stigmatization, self-identity threat, interpersonal deviance behavior, and moral disengagement. The results show that five common factors were extracted. The first common factor explained 20.014% of the total variance, less than 40% (Podsakoff et al., 2003). Therefore, there was no serious common deviance, and further analysis of the relevant data could be carried out.

## Results

Mplus is a powerful data processing software package that has been widely used in empirical research (e.g., Park et al., 2018; Bai et al., 2021). Therefore, we adopted Mplus 8.0 for the data analysis.

### Confirmatory Factor Analyses

We conducted confirmatory factor analysis (CFA) with Mplus 8.0 to examine whether our focal variables were distinctive constructs. The small sample size (*N* = 241) could have affected the validity of the fitting index. According to Marsh et al. (1998), item parceling could be used to increase the commonality between variables, to reduce random errors, and to enhance the fitting effect. At present, item parceling mainly includes the factor method (Rogers and Schmitt, 2004), correlation method, symmetric method, random method, and unique information method. This study chose the factor method to reduce the difference between groups because related research showed that this method would make the model parameter estimation more stable. In addition, the model fitting was better with this, rather than with other methods (Bandalos, 2008).

Factor analysis was carried out on all variables, arranged in descending order of factor load. The measurement indexes of the three variables of tour guide stigmatization, self-identity threat, and interpersonal deviance behavior were packaged into three observation indexes using the balance of high and low and then performing CFA. The result, as Table 1 shows, indicates that the proposed four-factor model showed a good fit to the data (χ^2^/df = 2.055, CFI = 0.940, TLI = 0.920, RMSEA = 0.066, SRMR = 0.058), and that it was significantly better than other models. The four-factor model has good discriminative validity and can test the relationship between various variables.

**TABLE 1:** Results of the confirmatory factor analysis.

Model	χ^2^	df	χ^2^/df	CFI	TLI	RMSEA	SRMR
1	121.224	59	2.055	0.940	0.920	0.066	0.058
2	276.414	62	4.458	0.792	0.739	0.120	0.092
3	516.642	64	8.073	0.562	0.466	0.171	0.140
4	731.043	65	11.247	0.355	0.226	0.206	0.167

*TGS, Tour guide stigmatization; SIT, Self-identity threat; IDB, Interpersonal deviance behavior; MD, Moral disengagement.*

*1. Four-factor model (TGS; SIT; IDB; MD).*

*2. Three-factor model (TGS + SIT; IDB; MD).*

*3. Tow-factor model (TGS + SIT + IDB; MD).*

*4. Signal-factor model (TGS + SIT + IDB + MD).*

### Descriptive Statistics

The descriptive statistics are shown in Table 2. Correlations indicated that tour guide stigmatization was positively related to interpersonal deviance behavior (*r* = 0.142, *p* < 0.05) and self-identity threat (*r* = 0.288, *p* < 0.01). The self-identity threat variable had a significantly positive effect on interpersonal deviance behavior (*r* = 0.206, *p* < 0.01). The correlation between the variables was consistent with the theoretical expectations.

**TABLE 2:** Descriptive statistics (*N* = 241).

Variables	Mean	SD	1	2	3	4	5	6	7	8	9	10	11
1. TGS	3.389	0.636											
2. SIT	2.964	0.682	0.288**										
3. IDB	2.340	0.725	0.142*	0.206**									
4. MD	2.186	0.599	–0.071	0.023	0.338**								
5. Gender	1.510	0.501	0.114	–0.116	–0.028	–0.082							
6. Age	33.870	7.010	0.072	0.132*	–0.116	−0.192**	−0.126*						
7. Education	3.490	0.895	0.132**	0.036	−0.150*	–0.071	0.082	–0.005					
8. BS	2.760	0.776	0.014	0.026	–0.023	–0.084	0.122	0.040	−0.197**				
9. OA	1.260	0.438	–0.089	–0.007	0.050	0.036	0.154*	–0.028	0.280**	−0.243**			
10. Tenure	4.240	1.294	0.136*	0.081	–0.118	−0.247**	−0.134**	0.525**	0.055	0.026	−0.161*		
11. OL	1.470	0.806	–0.105	0.048	0.152*	0.166*	–0.048	0.014	0.218**	−0.187**	0.185**	0.130*	
12. Income	3.070	1.375	0.055	–0.099	0.175**	–0.116	–0.095	0.119	−0.137*	–0.046	−0.210**	0.285**	0.090

BS, Business scope; OA, Occupational attribute; OL, Occupational level; *p < 0.05; **p < 0.01.

### Hypotheses Testing

#### The Effect of Tour Guide Stigmatization, Self-Identity Threat, and Interpersonal Deviance Behavior

The results of analysis on all variables showed (Table 3) that, in Model 3, the control variables of education, tenure, occupational level, and income were significantly related to interpersonal deviance behavior. Adding the tour guide stigmatization variable to Model 3 showed (Model 4) that tour guide stigmatization had a significantly positive effect on tour guides’ interpersonal deviance behavior (β = 0.219, *P* < 0.01). Therefore, Hypothesis 1 is supported. Model 2 showed that, among the control variables, only gender and income had a significant influence on self-identity threat, and tour guide stigmatization was positively correlated with self-identity threat (β = 0.319, *P* < 0.001). Therefore, Hypothesis 2 is supported. The results are shown for Model 5, demonstrating that self-identity threat has a significantly positive effect on tour guides’ interpersonal deviance behavior (β = 0.255, *P* < 0.001). The results provide support for Hypothesis 3.

**TABLE 3:** Hierarchical ridge regression results (*N* = 241).

	SIT		IDB					
	M1	M2	M3	M4	M5	M6	M7	M8
Gender	–0.113	−0.156*	–0.009	–0.039	0.020	–0.006	0.022	0.027
Age	0.113	0.107	–0.053	–0.507	–0.082	–0.079	–0.054	–0.038
Education	0.032	–0.023	−0.149*	−0.187**	−0.158*	−0.182**	−0.151*	−0.145*
OA	–0.013	0.024	–0.033	–0.007	–0.029	–0.012	–0.007	–0.019
OS	0.037	0.045	0.002	0.008	–0.007	–0.002	0.014	0.003
Tenure	0.034	–0.007	−0.184*	−0.213**	−0.193*	−0.221**	−0.146*	−0.164*
OL	0.028	0.071	0.235**	0.265***	0.228***	0.250***	0.191**	0.195**
Income	–0.128	−0.132*	0.227**	0.224**	0.260***	0.252***	0.267***	0.277***
TGS		0.319***		0.219**		0.152*	0.150*	0.167**
SIT					0.255***	0.208**	0.200**	0.219***
MD							0.290***	0.303***
SIT*MD								0.164**
*R* ^2^	0.047	0.140	0.128	0.171	0.190	0.209	0.282	0.307
△*R*^2^	0.014	0.107	0.098	0.139	0.158	0.174	0.247	0.271
*F*	1.432	4.189***	4.241***	5.311***	6.006***	6.070***	8.169***	8.431***

*p < 0.05; **p < 0.01, ***p < 0.001.

#### Test of Mediating Effect of Self-Identity Threat

We used the mediating effect test by Baron and Kenny (1986). First, Model 4 was used to test whether tour guide stigmatization significantly affected tour guides’ interpersonal deviance behavior. The result showed a significant positive effect (β = 0.219, *P* < 0.01). Model 2 was used to test whether tour guide stigmatization significantly affected self-identity threat, and the results showed a significant effect (β = 0.319, *P* < 0.01). Finally, tour guides’ interpersonal deviance behavior was regressed on tour guide stigmatization and self-identity threat (Model 6). The result showed that tour guide stigmatization still significantly affected self-identity threat (β = 0.152, *P* < 0.05) and self-identity threat still significantly affected tour guides’ interpersonal deviance behavior (β = 0.208, *P* < 0.01). However, the coefficient of Model 6 decreased compared with Model 4 (0.152 < 0.219), indicating that the positive effect of tour guide stigmatization on tour guides’ interpersonal deviance behavior was partly mediated by self-identity threat. This study used the bootstrapping approach for further verification and to ensure robustness. The results are shown in Table 4. The direct effect of tour guide stigmatization on tour guides’ interpersonal deviance behavior was 0.073 (95% Boot CI = [0.029, 0.318]). Their indirect effect was 0.076 (95% Boot CI = [0.028, 0.134]), indicating that the mediating effect of self-identity threat was significant, providing support for Hypothesis 4.

**TABLE 4:** The mediating role of self-identity threat (*N* = 241).

Path	Effect	SE	95% Boot CI		Mediate
			LLCI	ULCI	
TGS-IDB	Direct	0.073	0.029	0.318	Partly
TGS-SIT	Indirect	0.076	0.028	0.134	
TGS-SIT-IDB	Total	0.071	0.109	0.389	

#### Test of Moderating Effect of Moral Disengagement

**FIGURE 2:**
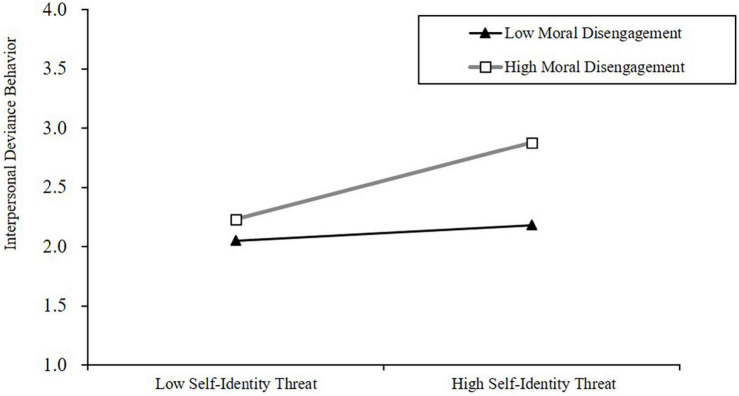
Moderating effect of MD.

Based on Model 7, we added the interaction effects between self-identity threat and moral disengagement on tour guides’ interpersonal deviance behavior (Model 8). The results showed that the interaction between the independent (self-identity threat) and the moderator variable (moral disengagement) had a significant effect on tour guides’ interpersonal deviance behavior (β = 0.164, *P* < 0.01). To examine the moderating effect of moral disengagement on self-identity threat and tour guides’ interpersonal deviance behavior more closely, we conducted a significance analysis of simple slopes at high and low levels of moral disengagement (1 SD above and below the mean value). The results (Figure 2) show that when moral disengagement is low, self-identity threat has no significant impact on tour guides’ interpersonal deviance behavior (β = 0.043, *P* = ns). However, when moral disengagement is high, self-identity threat has a significant impact on tour guides’ interpersonal deviance behavior (β = 0.271, *P* < 0.01). Therefore, moral disengagement positively moderated the relationship between self-identity threat and tour guides’ interpersonal deviance behavior. Hypothesis 5 is supported.

#### Test of Moderated Mediating Effect

Bootstrap sampling was used in this study to verify the effect of tour guide stigmatization on tour guides’ interpersonal deviance behavior and to determine whether the mediating effect and the moderating effect occur simultaneously, that is, whether there is a moderated mediating effect. The results are shown in Table 5. Under the lower level of moral disengagement, the estimated value of the indirect effect of tour guide stigmatization on tour guides’ interpersonal deviance behavior (*via* self-identity threat) was non-significant (β = 0.034, 95% Boot CI = [−0.051, 0.086]). At a high level of moral disengagement, the estimated value of the indirect effect of tour guide stigmatization on tour guides’ interpersonal deviance behavior (*via* self-identity threat) was significant (β = 0.044, 95% Boot CI = [0.062, 0.232]). The difference between two conditional indirect effects was also significant (β = 0.059, 95% Boot CI = [0.011, 0.245]). The results reveal that moral disengagement moderated the indirect effect of tour guide stigmatization on tour guides’ interpersonal deviance behavior *via* self-identity threat. Thus, Hypothesis 6 is supported.

**TABLE 5:** Moderated mediation effect test results (*N* = 241).

Moral disengagement	Indirect Effect	SE	95% Boot CI	
			LLCI	ULCI
Low(−SD)	0.008	0.034	−0.051	0.086
High(+SD)	0.151	0.044	0.062	0.232
Difference	0.143	0.059	0.011	0.245

## Discussion

This study focused on the mechanism of tour guide stigmatization and tour guides’ interpersonal deviance behavior, specifically discussing the relationship between tour guide stigmatization, self-identity threat, moral disengagement, and interpersonal deviance behavior. Based on sample data from 241 tour guides, our findings suggest that tour guide stigmatization can result in tour guides’ interpersonal deviance behavior and self-identity threat. When perceiving stigma, tour guides perceive self-identity threat, which in turn increases tour guides’ interpersonal deviance behavior. Moreover, this study highlights that moral disengagement moderates the direct effect of self-identity threat on tour guides’ interpersonal deviance behavior, as well as the indirect effect of tour guide stigmatization on tour guides’ interpersonal deviance behavior through self-identity threat, making these effects stronger for tour guides.

### Theoretical Implications

First, this study expands the understanding of tour guide stigmatization and responds to calls from industry and academia for more research on tour guide stigmatization (Li et al., 2020; Frost et al., 2021). Past research on tour guide stigmatization mainly focused on the antecedents (e.g., deceiving and insulting tourists; Li et al., 2020), but paid little attention to the influence of tour guide stigmatization from an interpersonal perspective. Only Yang and Liu (2011) elaborated on the phenomenon through a qualitative approach. Therefore, this study expands the research on tour guide stigmatization from content and approaches. By constructing a theoretical model of tour guide stigmatization and tour guides’ interpersonal deviance behavior, and verifying the hypotheses proposed, this study not only adds an application case for empirical research on tour guide stigmatization but also provides theoretical support for understanding the interpersonal behavior of tour guides who are deeply disturbed by professional stigma. This study adds to the knowledge of the influence of stigmatization on tour guide service behaviors, enriches tour guide stigmatization literature, and provides a direction for further research on the fundamental problem of tour guide stigmatization.

Second, this study introduced self-identity threat theory to the research on tour guide stigmatization to explain the influence of tour guide stigmatization on tour guides’ interpersonal deviance behavior. It provides novel insights into the theoretical mechanism of the influence of tour guide stigmatization on interpersonal deviance behavior. The actual situation showed that the interpersonal deviance behavior of tour guides, such as insulting and cheating tourists, was an important reason for tour guide stigmatization. However, the study of occupational stigma found that stigmatization could lead to interpersonal deviance behavior (Levin and Van Laar, 2006). To eliminate tour guide stigmatization, it is very important to study the mechanism between tour guide stigmatization and tour guides’ interpersonal deviance behavior. By drawing on self-identity threat theory and examining the mediative effect of self-identity threat, we found that tour guide stigmatization not only directly causes tour guides’ interpersonal deviance behavior but also indirectly causes tour guides’ interpersonal deviance behavior through self-identity threat. These results further support the negative impact of occupational stigmatization on self-identity and support the rationality of the two-stage process of identity threat theory.

Third, the study expanded self-identity threat theory from a new perspective and clarified the boundary conditions for the effect of tour guide stigmatization *via* self-identity threat on alleviating tour guides’ interpersonal deviance behavior. Some of the moderating effects between self-identity threat and interpersonal deviance behaviors had already been identified in the research literature. For example, an individual deviance identity will positively regulate the relationship between self-identity threat and interpersonal deviance behaviors (Kaplan and Lin, 2000). However, this effect had not been explored from a moral perspective. Unlike previous studies that demonstrated moral disengagement as a mediator (Liu et al., 2021), we argue that it is a key underlying mechanism that moderates the effect of stigmatization on tour guides’ negative behaviors. By investigating the moderating role of moral disengagement, our findings indicate that lower moral disengagement was not significantly responsive to the effect between self-identity threat and tour guides’ interpersonal deviance behavior. In contrast, the moderating effect of high moral disengagement was significant. The findings advance the understanding of the role of tour guide moral disengagement on the impact of self-identity threat on tour guides’ interpersonal deviance behavior and suggest implications for how to train tour guides to weaken the impact of self-identity threat on deviance behavior.

### Practical Implications

Our study has several implications for tour guides and tourism organizations. First, enhancing the self-identity of tour guides alleviates self-identity threat. The results of the moderated mediating effect showed that it is very important to mitigate the self-identity threat caused by stigmatization. Deaux (1993) believed that an identity negotiation strategy could be adopted to mitigate self-identity threat. The strategy included not only the negotiation with the inner self to meet the needs of self-esteem but also included the negotiation necessary to gain social approval. Specifically, the relevant management departments of tour guides need to provide conditions and signals to strengthen career self-affirmation and deepen the significance of the work of tour guides by, for example, emphasizing the positive attributes of the work. Simultaneously, it is necessary to formulate certain measures or carry out activities to try to change the meaning and value that society assigns to tour guides, such as actively promoting the importance of tour guides in the travel process and making good use of the news media to report positive images of tour guides.

Moreover, improving the moral level of tour guides will reduce the occurrence of interpersonal deviance behavior among them. Moral disengagement plays an important role in promoting the influence of self-identity threat on tour guides’ interpersonal deviance behavior. The results of this study show that to reduce tour guides’ interpersonal deviance behavior, we must pay attention to improving the level of tour guide moral identity, thereby reducing their moral disengagement level. First, we need to conduct strict screening and select tour guides with a certain level of education, ability, and political integrity. This would improve the qualification levels of tour guides. Second, tour guides’ self-discipline awareness should be strengthened, thereby ensuring compliance with professional ethics and adherence to social and moral standards. Meanwhile, self-discipline management in the industry should be strengthened by establishing an industry mechanism and cultivating a sense of responsibility among tour guides. In addition, the establishment of a moral evaluation mechanism for reward and punishment is worthy of consideration.

Finally, the vocational training of tour guides and the optimization of their service response ability should be prioritized. Under the combined influence of self-identity threat and moral disengagement, tour guides who perceived stigma were more likely to engage in interpersonal deviance behavior. The results show that tour guides still lack suitable ways to interact with hostile tourists. In this regard, tour guide management and training departments should actively discuss and teach tour guides how to deal properly with conflicts with tourists in stigmatization situations (Ashforth et al., 2007) rather than using interpersonal deviance behavior to resist or retaliate, thereby further aggravating tourists’ negative stereotypes of tour guides.

### Limitations and Future Research

Several limitations of our study need to be noted for future research. First, the data collected in this research belong to cross-sectional and subjective reporting. There are limitations in the inferred causality between variables (Evans, 1985), and there are concerns about the accuracy of self-reported data. In addition, the problem of common method bias cannot be completely avoided. Therefore, future research should collect longitudinal data to verify the validity of the causal relationship of variables and measure the variables from a third-party perspective (e.g., tourists). It should also use tour guide interviews, experimental design, and other methods to reduce common method bias.

Second, this article is based on identity threat theory and models constructed using tour guide stigmatization, self-identity threat, and interpersonal deviance behavior to reveal the mediative effect of self-identity threat. However, in view of the close relationship between individual emotions and behavior (Lawrence, 1974), future research could use emotion as an intermediary to explore the mechanism between tour guide stigmatization and interpersonal deviance behavior.

Third, research on occupational stigma and deviance behavior has shown that, in addition to the individual moral level, professional group support, organizational support, and family support could be used as moderators (Schwarzer and Weiner, 1991). These factors could have an important impact on alleviating stigma and reducing interpersonal deviance behavior. Future research could further verify the moderating effect of other individual variables and could also analyze group variables.

Finally, although this article verified the relationship between tour guide stigmatization and tour guides’ interpersonal deviance behavior, it is not rich enough to explain all outcomes of tour guide stigmatization. On the one hand, the impact of stigma on interpersonal interaction not only involves hostile behaviors, such as interpersonal deviance but also withdrawal behavior (Newheiser et al., 2015). On the other hand, stigmatization affects not only the interpersonal behavior of tour guides but also the behavior between tour guides and their organizations. In addition, research on occupational stigma has found that not all group effects of stigma are negative. Some effects are likely to deepen the identity of the inner group and enhance the group’s unity (Crabtree et al., 2010). Therefore, future research could explore tour guide stigmatization in terms of withdrawal behavior, organizational deviance behavior, and inner-group behavior.

## Data Availability Statement

The raw data supporting the conclusions of this article will be made available by the authors, without undue reservation.

## Ethics Statement

Ethical review and approval was not required for the study on human participants in accordance with the local legislation and institutional requirements. All the participants provided their written informed consent to participate in this study.

## Author Contributions

AD, WL, and AL contributed to the conception and design of the study. AL organized the database and wrote the first draft of the manuscript. YZ performed the statistical analysis. AD, WL, and KG wrote sections of the manuscript. All authors contributed to manuscript revision, read, and approved the submitted version.

## Conflict of Interest

The authors declare that the research was conducted in the absence of any commercial or financial relationships that could be construed as a potential conflict of interest.

## Publisher’s Note

All claims expressed in this article are solely those of the authors and do not necessarily represent those of their affiliated organizations, or those of the publisher, the editors and the reviewers. Any product that may be evaluated in this article, or claim that may be made by its manufacturer, is not guaranteed or endorsed by the publisher.
